# Supplementary figures and images for: Loss of the m6A methyltransferase METTL3 in monocyte-derived macrophages ameliorates Alzheimer’s disease pathology in mice
Source: PLoS Biol. 2023 Mar 7;21(3):e3002017. doi: 10.1371/journal.pbio.3002017 (PMC9990945; doi:10.1371/journal.pbio.3002017)

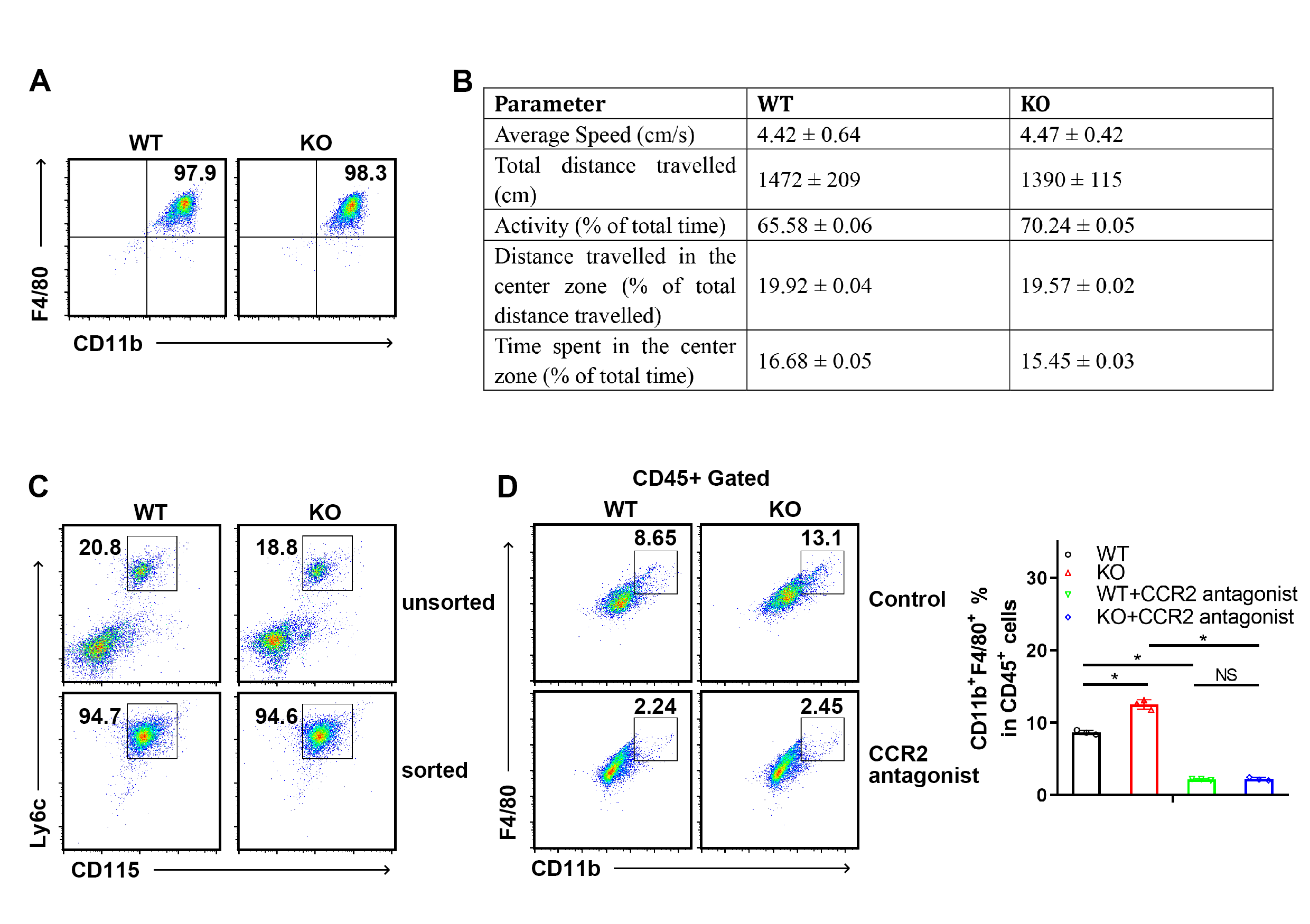

Supplement: S1 Fig — (A) Flow cytometry of the surface CD11b and F4/80 expression of primary cultured bone marrow-derived macrophages. (B) Aβ-injected WT and KO mice were assessed in the open field test. Data are presented as a mean ± SEM. (C) The purity of isolated monocytes was checked by flow cytometry. (D) Flow cytometry analysis of brains from Aβ-induced WT or KO mice injected with CCR2 antagonist PF-4136309 (2 mg/kg). P < 0.05 (*). NS means no significant difference. Underlying data can be found in S1 Data. Aβ, amyloid beta; KO, knockout; WT, wild type. (TIF) [file pbio.3002017.s001.tif]

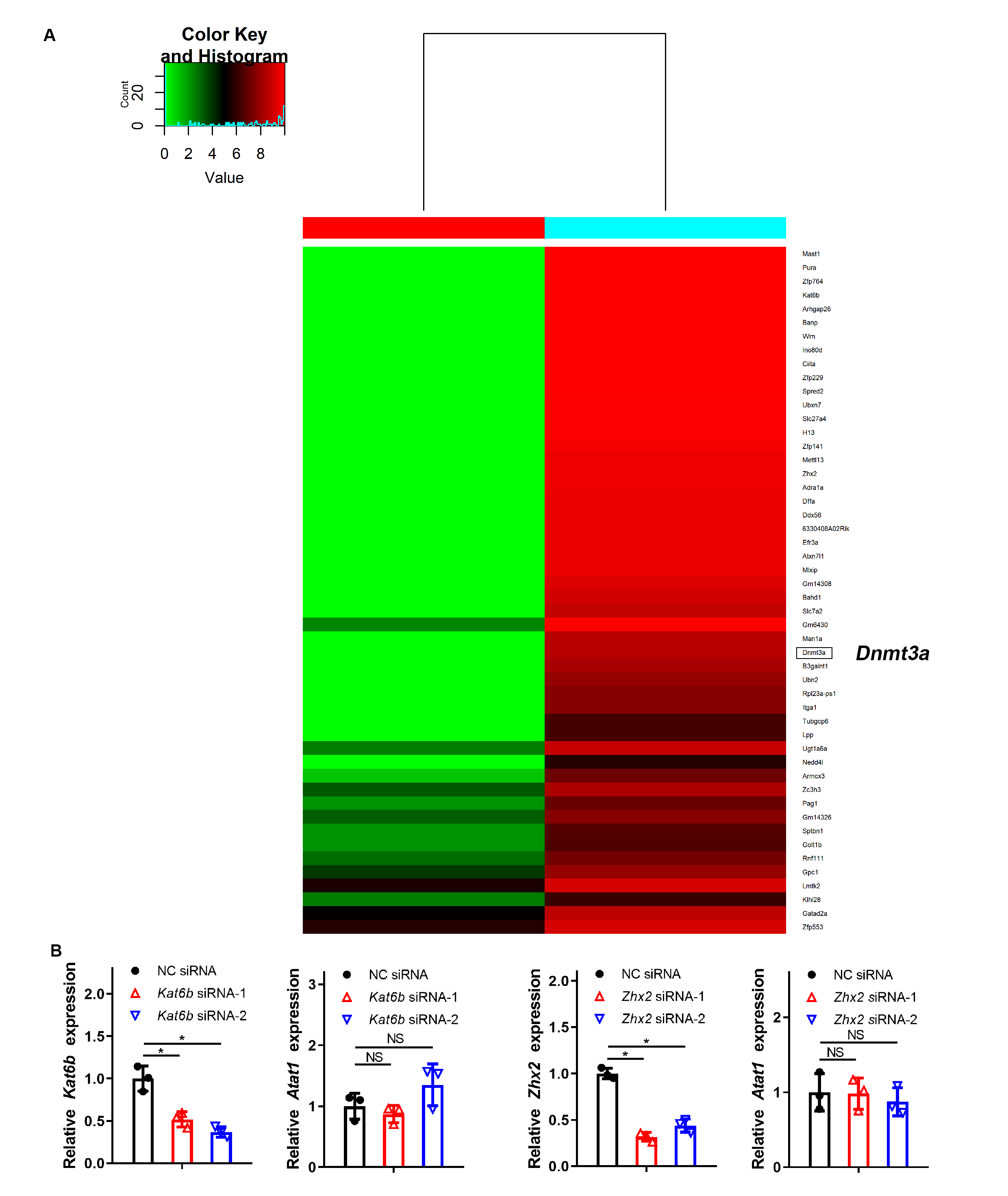

Supplement: S2 Fig — (A) The ranking of top 50 m6A down-regulated genes in KO BMDMs. (B) The expression of Atat1 was measured by qRT-PCR in BMDMs with Kat6b or Zhx2 knockdown. Data in the figure are shown as mean ± SD. P < 0.05 (*). NS means no significant difference. Underlying data can be found in S1 Data. KO, knockout; qRT-PCR, quantitative reverse transcription PCR. (TIF) [file pbio.3002017.s002.tif]

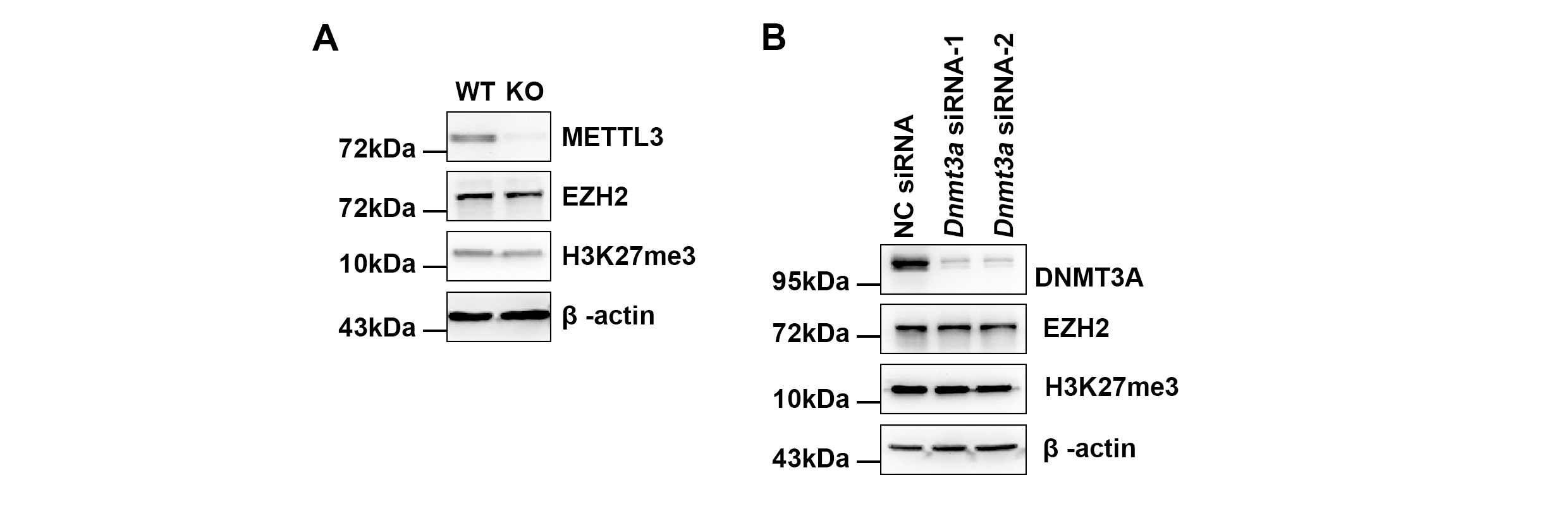

Supplement: S3 Fig — (A) Immunoblotting analysis of the indicated proteins in WT and KO BMDMs. (B) Immunoblotting analysis of the indicated proteins in BMDMs transfected with NC siRNA or Dnmt3a siRNA. Underlying data can be found in S1 Data. KO, knockout; NC, negative control; WT, wild type. (TIF) [file pbio.3002017.s003.tif]

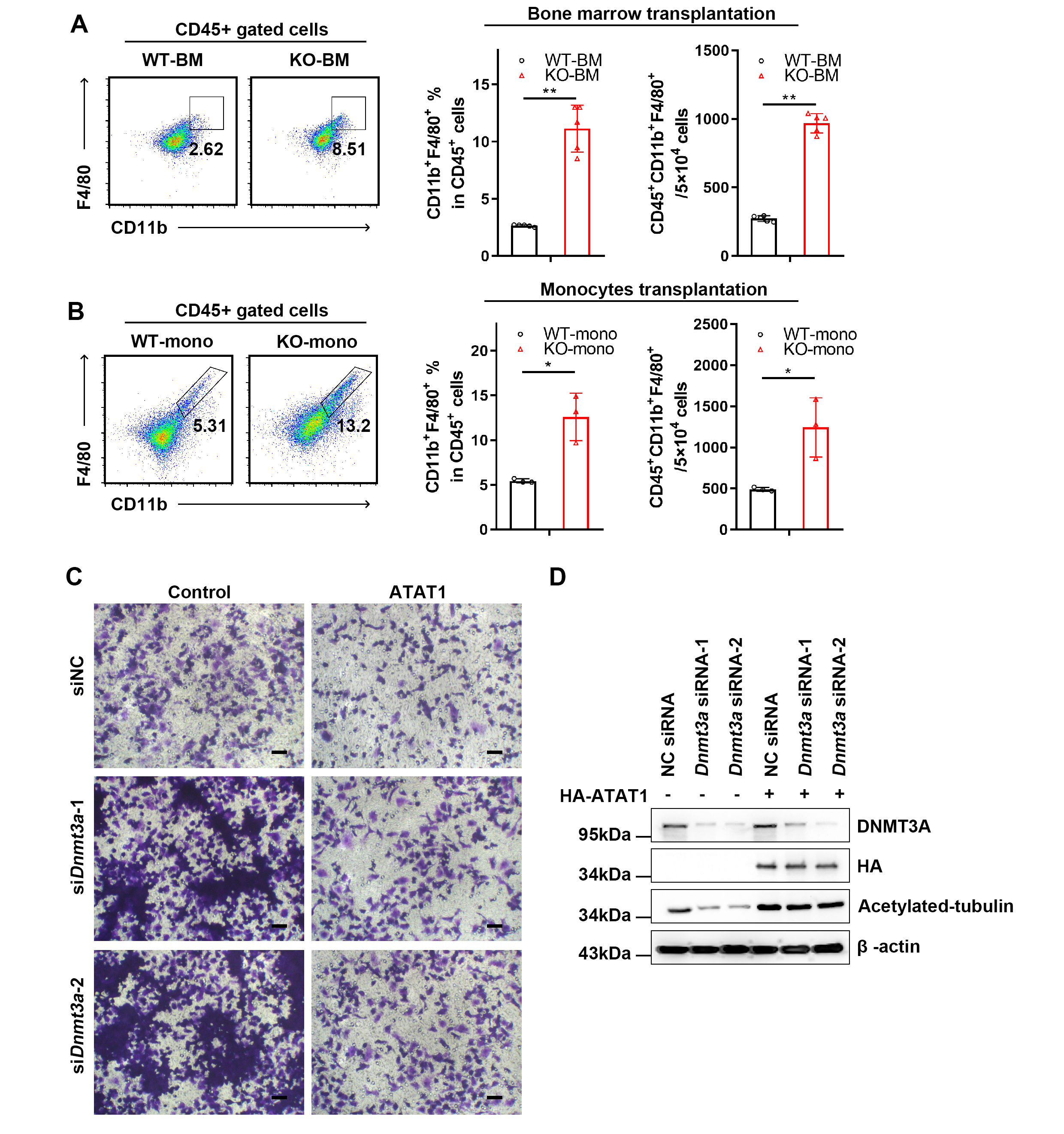

Supplement: S4 Fig — (A) Flow cytometry analysis of brains from Aβ-induced mice transferred with WT and KO BM. (B) Flow cytometry analysis of brains from Aβ-induced mice (CD45.1) transferred with WT or KO monocytes (CD45.2). (C) Transwell assays were performed to determine the migration of ATAT1-overexpressing BMDMs transfected with NC siRNA or Dnmt3a siRNA. Scale bars: 30 μm. (D) Immunoblotting analysis of the indicated proteins in ATAT1-overexpressing BMDMs transfected with NC siRNA or Dnmt3a siRNA. Data in the figure are shown as mean ± SD. P < 0.05 (*), P < 0.01 (**). NS means no significant difference. Underlying data can be found in S1 Data. Aβ, amyloid beta; KO, knockout; NC, negative control; WT, wild type. (TIF) [file pbio.3002017.s004.tif]

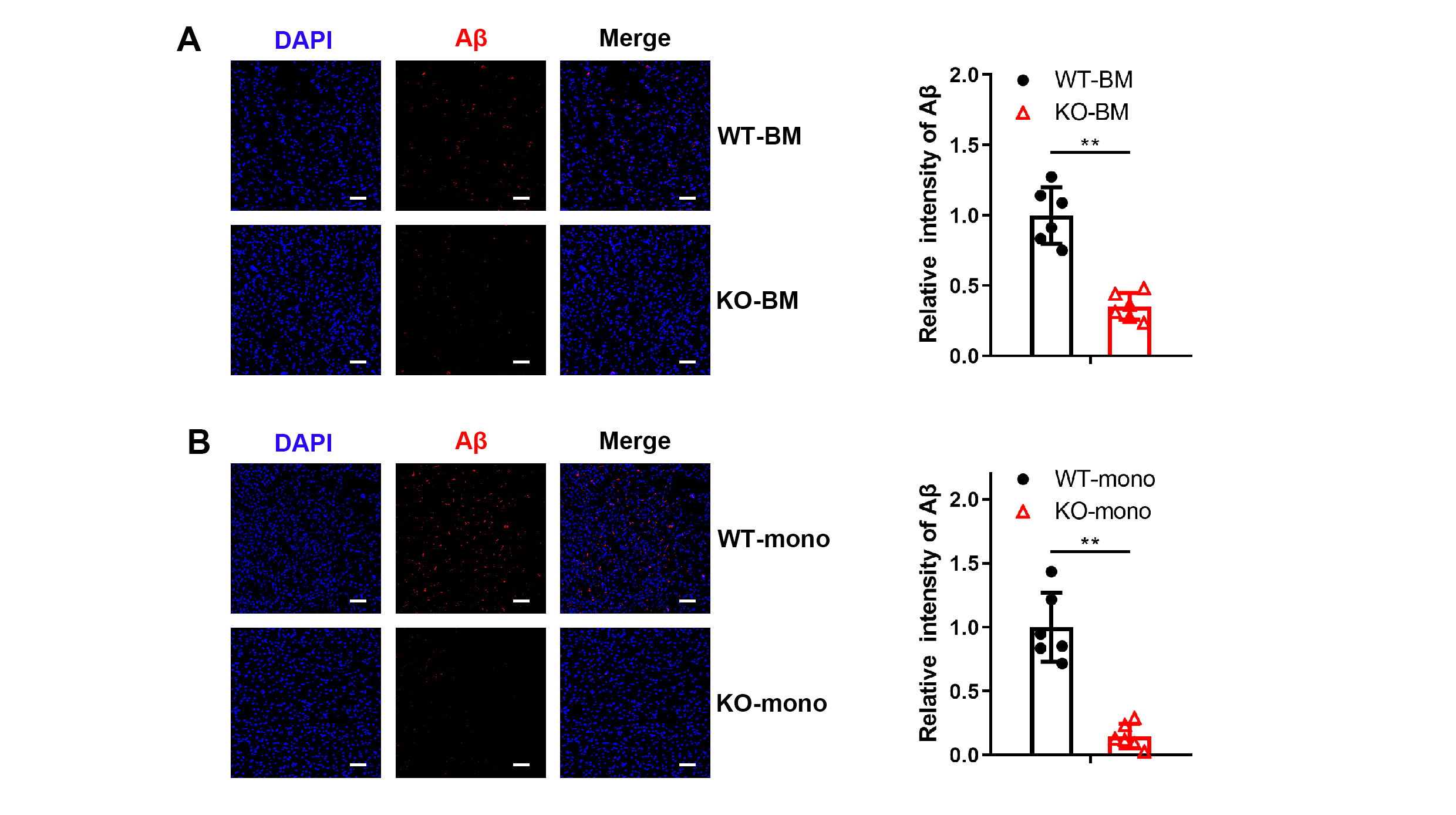

Supplement: S5 Fig — (A) Representative fluorescence micrographs and quantitative analysis of brains from Aβ-induced mice transferred with WT and KO BM. (B) Representative fluorescence micrographs and quantitative analysis of brain from Aβ-induced mice transferred with WT and KO monocytes. Scale bars: 50 μm. Data in the figure are shown as mean ± SD. P < 0.01 (**). Underlying data can be found in S1 Data. Aβ, amyloid beta; KO, knockout; WT, wild type. (TIF) [file pbio.3002017.s005.tif]

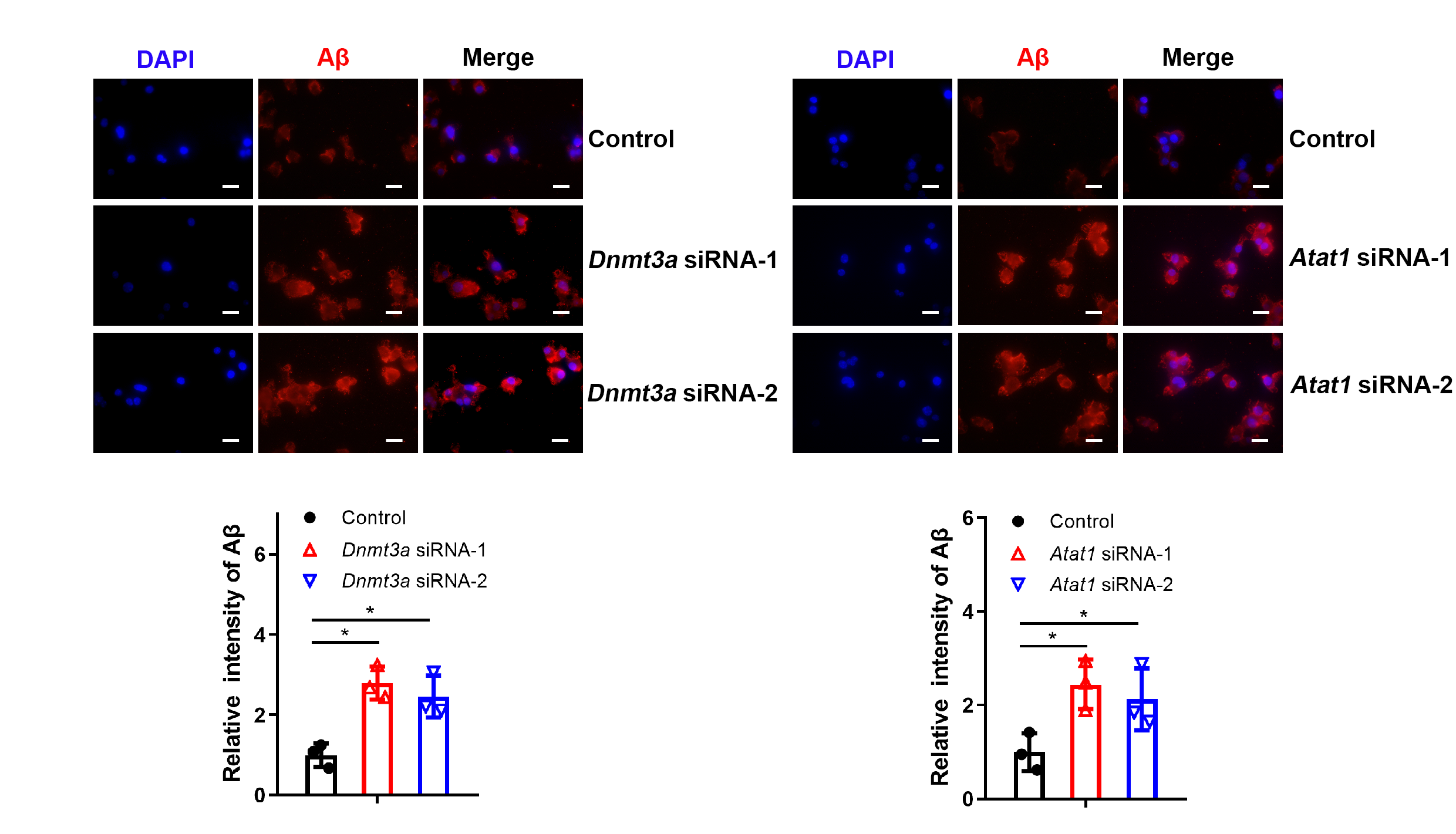

Supplement: S6 Fig — (A) Representative fluorescent micrographs and quantitative analysis of Aβ uptake for 24 h in BMDMs transfected with NC siRNA or Dnmt3a siRNA and stained with Aβ antibody. Scale bars: 10 μm. (B) Representative fluorescent micrographs and quantitative analysis of Aβ uptake for 24 h in BMDMs transfected with NC siRNA or Atat1 siRNA and stained with Aβ antibody. Scale bars: 10 μm. Data in the figure are shown as mean ± SD. P < 0.05 (*). Underlying data can be found in S1 Data. Aβ, amyloid beta; NC, negative control. (TIF) [file pbio.3002017.s006.tif]
