# Supplementary material for: Loss of the m6A methyltransferase METTL3 in monocyte-derived macrophages ameliorates Alzheimer’s disease pathology in mice
Source: PLoS Biol. 2023 Mar 7;21(3):e3002017. doi: 10.1371/journal.pbio.3002017 (PMC9990945; doi:10.1371/journal.pbio.3002017)
Supplement: S1 Table — (DOCX) [file pbio.3002017.s007.docx]

**S1 Table.**  **siRNA sequences used in this manuscript.**

|  | sense（5'-3'） | antisense（5'-3'） |
| --- | --- | --- |
| *Dnmt3a*-siRNA-1（Mus musculus） | GCAUCCACUGUGAAUGAUATT | UAUCAUUCACAGUGGAUGCTT |
| *Dnmt3a* -siRNA-2（Mus musculus） | GCAGAACAAGCAGAUGAUUTT | AAUCAUCUGCUUGUUCUGCTT |
| *Atat1*-siRNA-1（Mus musculus） | GCAACCGGCACGUUAUUUATT | UAAAUAACGUGCCGGUUGCTT |
| *Atat1*-siRNA-2（Mus musculus） | CCCACAGGUGAACAACUUUTT | AAAGUUGUUCACCUGUGGGTT |
| *Ythdf1*-siRNA-1（Mus musculus） | CCCGUAUCUCACUACCUAUTT | AUAGGUAGUGAGAUACGGGTT |
| *Ythdf1*-siRNA-2（Mus musculus） | GGACAUUGGUACUUGGGAUTT | AUCCCAAGUACCAAUGUCCTT |
| *Mettl3*-siRNA-1（Mus musculus） | CUGCACUUCAGACGAAUUATT | UAAUUCGUCUGAAGUGCAGTT |
| *Mettl3*-siRNA-2（Mus musculus） | CAGUCAUAAACCAGAUGAATT | UUCAUCUGGUUUAUGACUGTT |
| *Kat6b*-siRNA-1（Mus musculus） | GCCGAUCCCAUUCCAAUAUTT | AUAUUGGAAUGGGAUCGGCTT |
| *Kat6b*-siRNA-2（Mus musculus） | GCGGUGUUGAUCUCACAAUTT | AUUGUGAGAUCAACACCGCTT |
| *Zhx2*-siRNA-1（Mus musculus） | CCCGGAAGAAGAUGUUUAATT | UUAAACAUCUUCUUCCGGGTT |
| *Zhx2*-siRNA-2（Mus musculus） | GCGGUUAUGAGUGCAAAUATT | UAUUUGCACUCAUAACCGCTT |
| *Ezh2*-siRNA（Mus musculus ） | GAAAGAUCUAGAGGAUAAUTT | AUUAUCCUCUAGAUCUUUCTT |
