# Supplementary material for: Loss of the m6A methyltransferase METTL3 in monocyte-derived macrophages ameliorates Alzheimer’s disease pathology in mice
Source: PLoS Biol. 2023 Mar 7;21(3):e3002017. doi: 10.1371/journal.pbio.3002017 (PMC9990945; doi:10.1371/journal.pbio.3002017)
Supplement: S2 Table — (DOCX) [file pbio.3002017.s008.docx]

| **S2 Table. qRT-PCR primers used in this manuscript.** | | |
| --- | --- | --- |
| *Mettl3*（Mus musculus ） | Forward primer | CCAGCACAACATCTGTGGC |
|  | Reverse primer | CGCTTTACCTCAATCAACTCCTG |
| *Ythdf1*（Mus musculus ） | Forward primer | ACAGTTACCCCTCGATGAGTG |
|  | Reverse primer | GGTAGTGAGATACGGGATGGGA |
| *Atat1*（Mus musculus ） | Forward primer | CTGGGGAGGAGCCTACACA |
|  | Reverse primer | GACCCTGGCGTTCATTATGTC |
| *Dnmt3a*（Mus musculus ） | Forward primer | GAGGGAACTGAGACCCCAC |
|  | Reverse primer | CTGGAAGGTGAGTCTTGGCA |
| *Hdac6*（Mus musculus ） | Forward primer | TCCACCGGCCAAGATTCTTC |
|  | Reverse primer | CAGCACACTTCTTTCCACCAC |
| *Kat6b*（Mus musculus ） | Forward primer | AGAAGAAAAGGGGTCGTAAACG |
|  | Reverse primer | GTGGGAATGCTTTCCTCAGAA |
| *Zhx2*（Mus musculus ） | Forward primer | ATGGCAAGCAAACGGAAATCT |
|  | Reverse primer | TCCTTTGTCACATCGGACTGT |
| *Sirt2*（Mus musculus ） | Forward primer | GCCTGGGTTCCCAAAAGGAG |
|  | Reverse primer | GAGCGGAAGTCAGGGATACC |
| *Ezh2*（Mus musculus ） | Forward primer | AGTGACTTGGATTTTCCAGCAC |
|  | Reverse primer | AATTCTGTTGTAAGGGCGACC |
| Chip-Mus-*Atat1*-up1k | Forward primer | AGGCACTGTGAAACCATGGT |
|  | Reverse primer | ATTGTGCGGGACCAGACA |
| Chip-Mus-*Atat1*-up2k | Forward primer | TGTAGACCAGGCTGGCCT |
|  | Reverse primer | CTCTGACAAAAGGGACATAT |
| Chip-Mus-*Atat1*-up3k | Forward primer | AAGCACAGACCCACTTGC |
|  | Reverse primer | AAGGCCAGCAAGTGCTAC |
| Chip-Mus-*Atat1*-up4k | Forward primer | CTTGAAGCACATCCTAGAG |
|  | Reverse primer | AGACACATCGGTGGAGTT |
| Chip-Mus-*Atat1*-up5k | Forward primer | GTCTTGATTTCAGAATACCAC |
|  | Reverse primer | CTCACAATACATGTCTTTCG |
