# Supplementary material for: Loss of the m6A methyltransferase METTL3 in monocyte-derived macrophages ameliorates Alzheimer’s disease pathology in mice
Source: PLoS Biol. 2023 Mar 7;21(3):e3002017. doi: 10.1371/journal.pbio.3002017 (PMC9990945; doi:10.1371/journal.pbio.3002017)

Original images (blots) of Fig 2A

Membranes scanned using Amersham Imager 680 or Tanon 5500

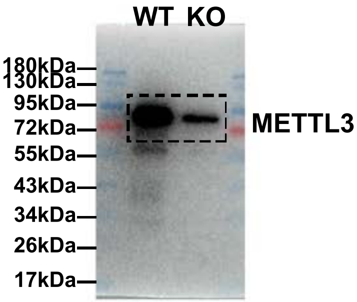

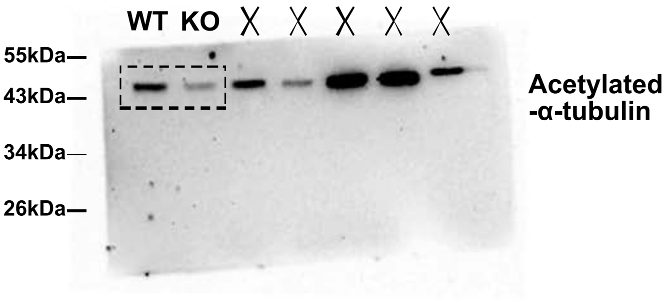

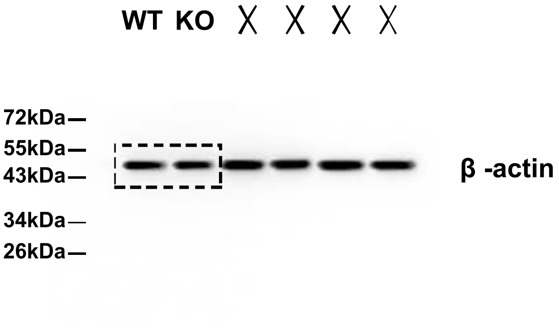

Original images (blots) of Fig 2B

Membranes scanned using Amersham Imager 680

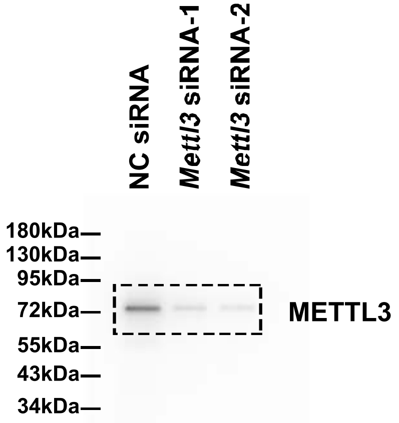

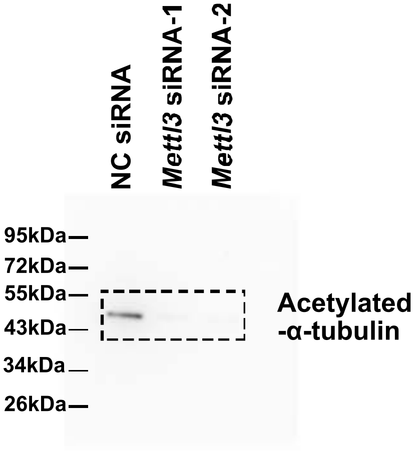

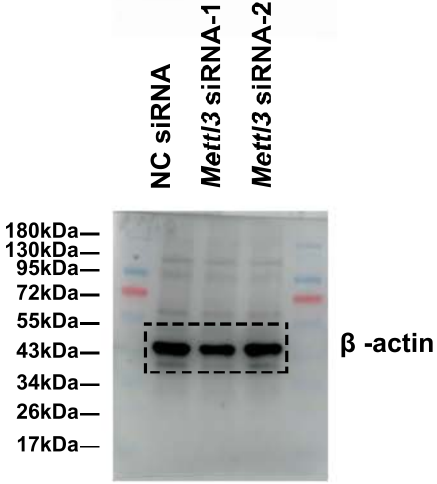

Original images (blots) of Fig 2C

Membranes scanned using Tanon 5500

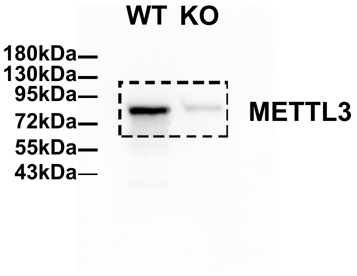

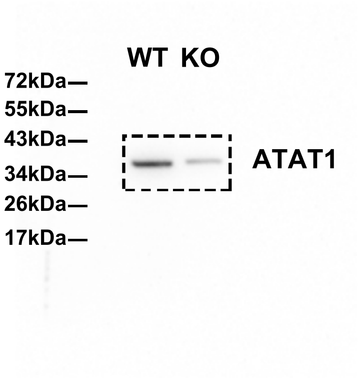

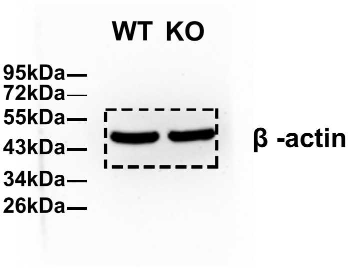

Original images (blots) of Fig 2D

Membranes scanned using Tanon 5500

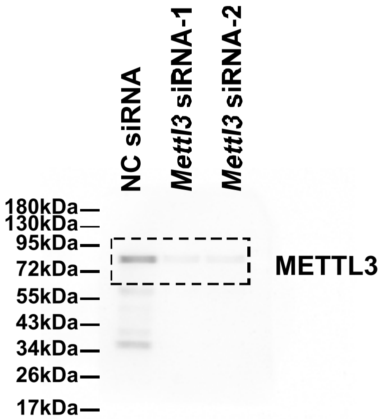

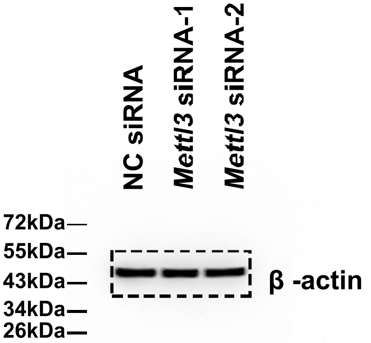

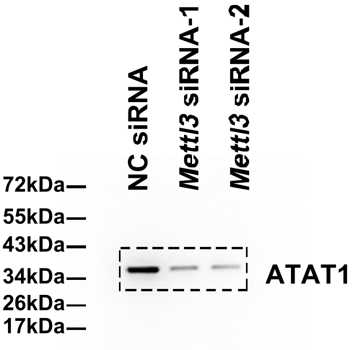

Original images (blots) of Fig 3E

Membranes scanned using Tanon 5500

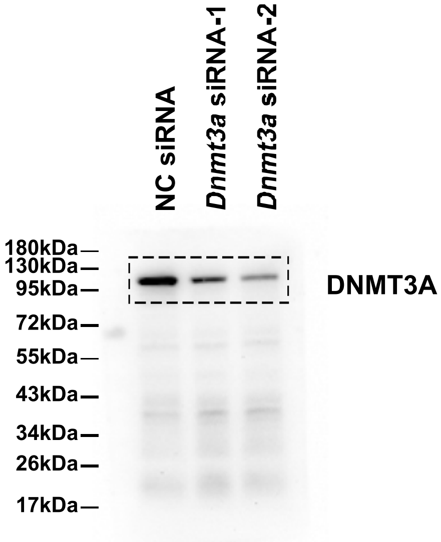

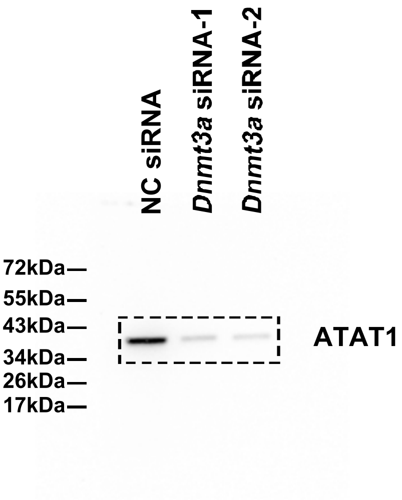

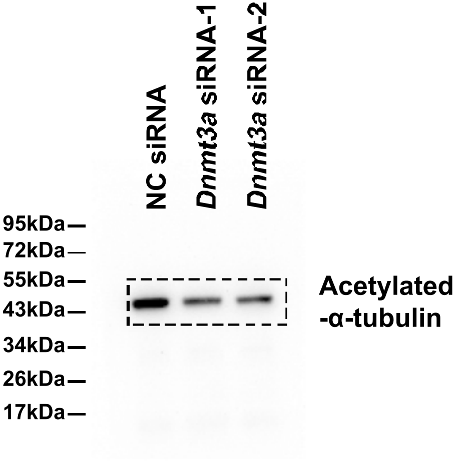

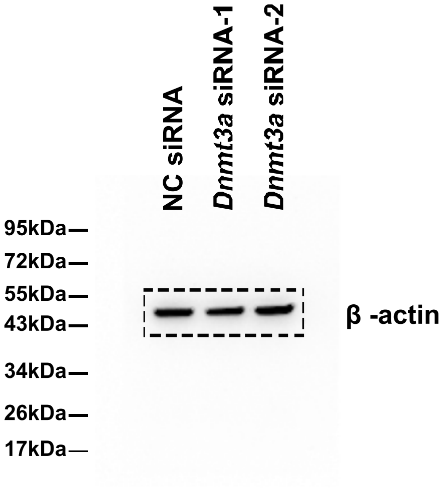

Original images (blots) of Fig 4B

Membranes scanned using Tanon 5500

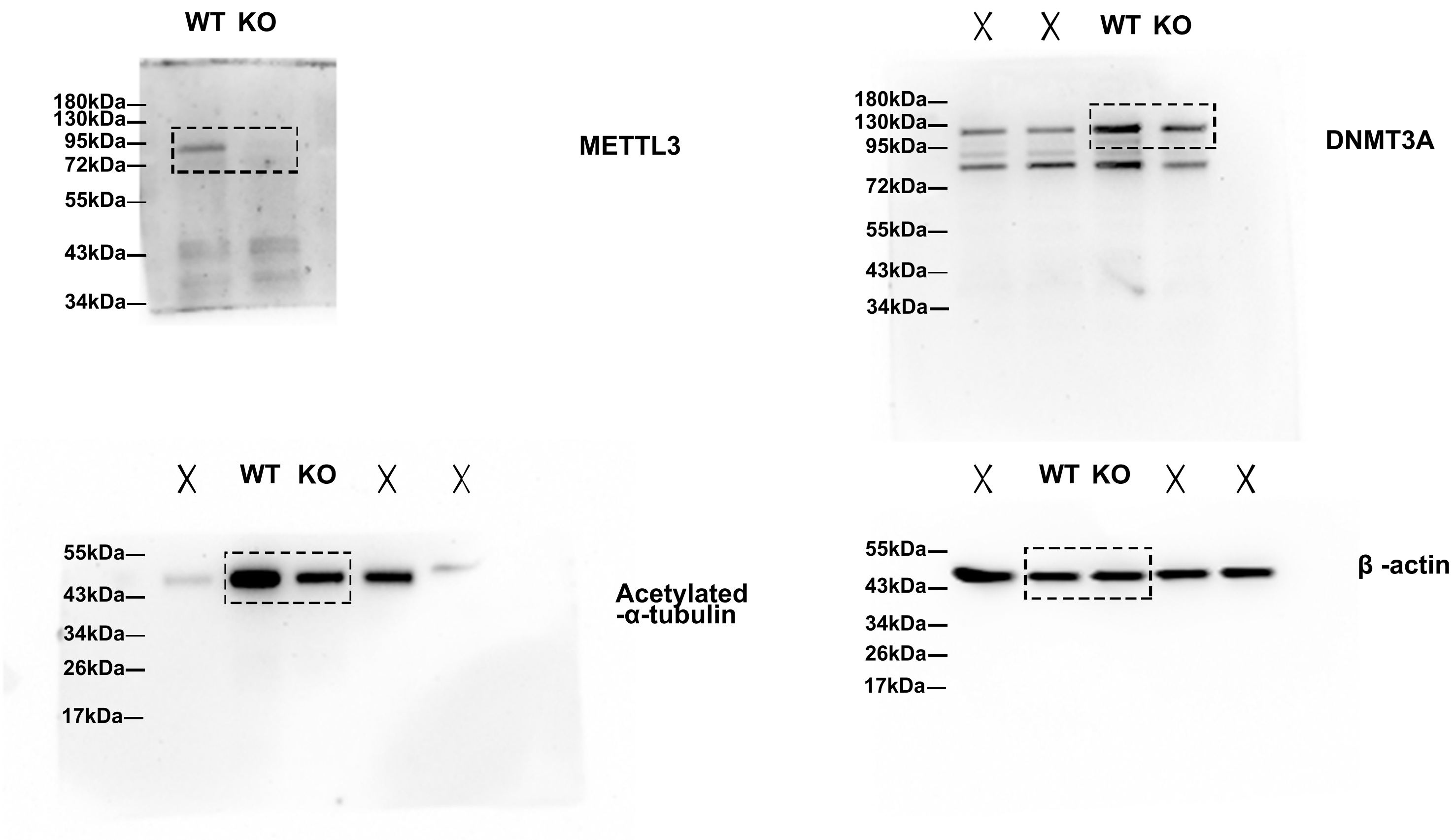

Original images (blots) of Fig 4C

Membranes scanned using Tanon 5500

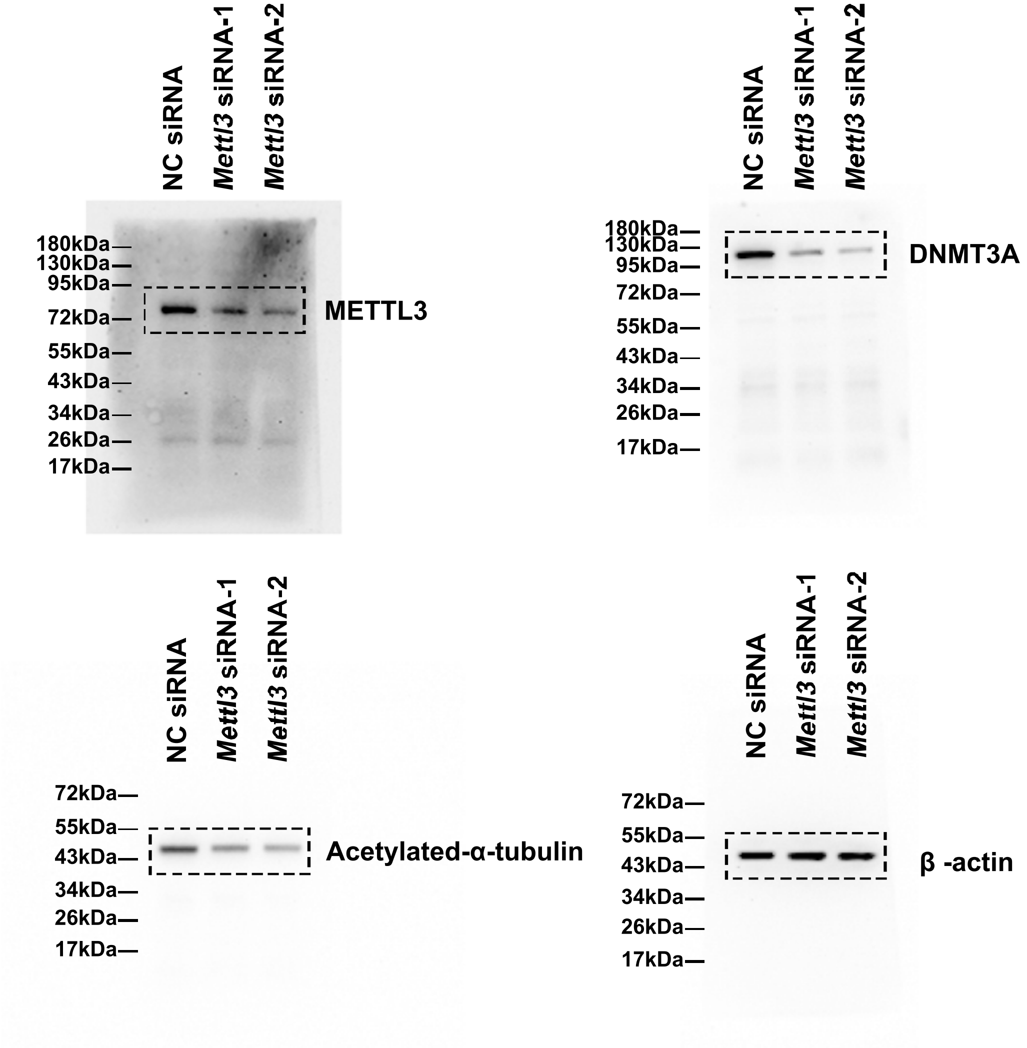

Original images (blots) of Fig 4G

Membranes scanned using Tanon 5500

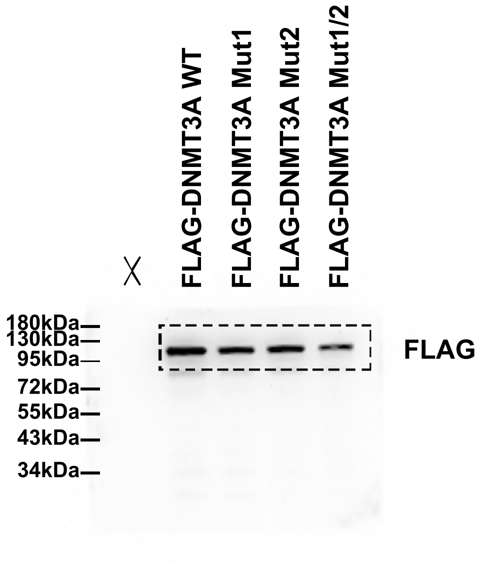

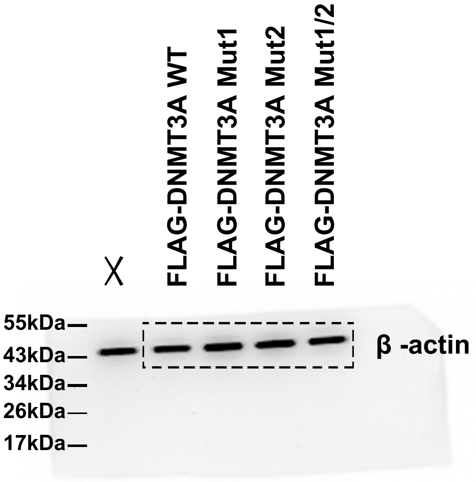

Original images (blots) of Fig 4J

Membranes scanned using Tanon 5500

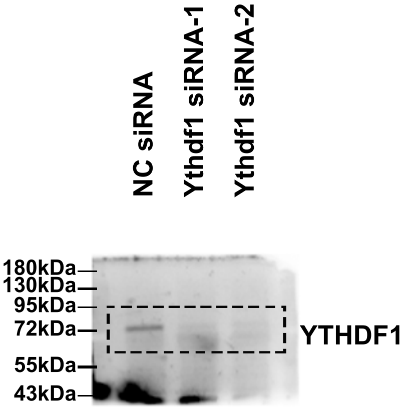

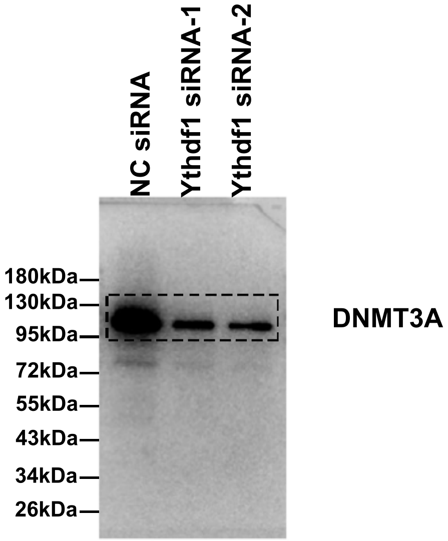

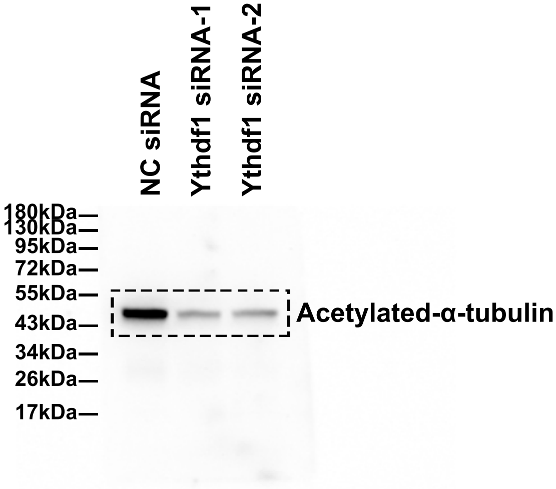

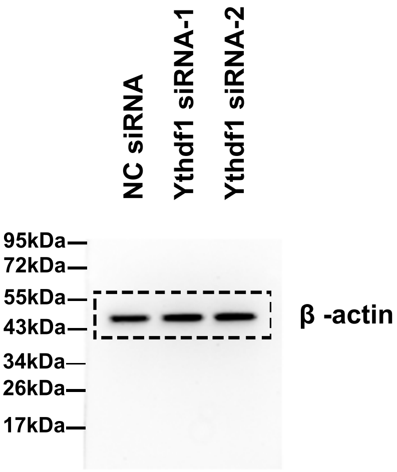

Original images (blots) of Fig 5D

Membranes scanned using Tanon 5500

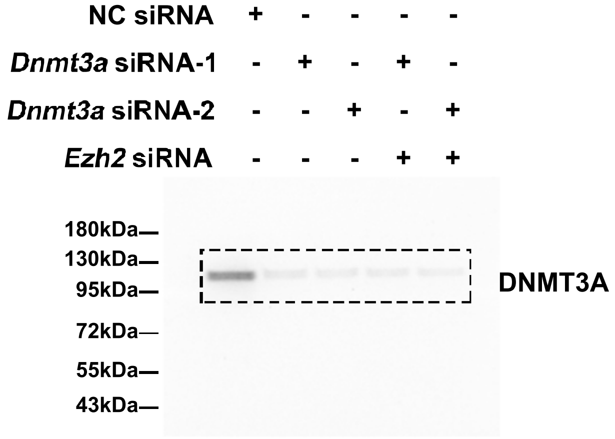

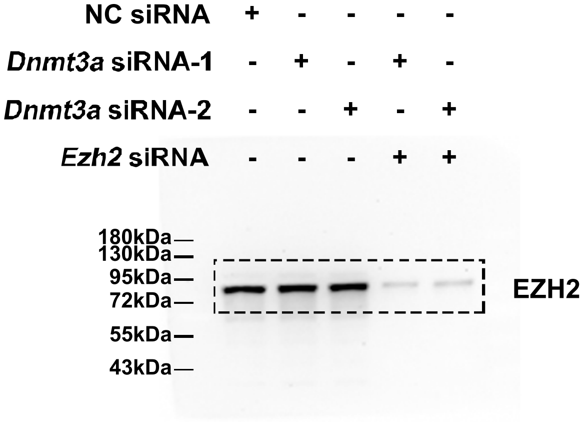

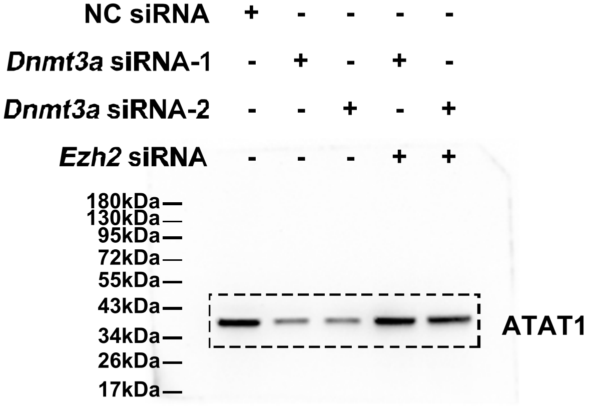

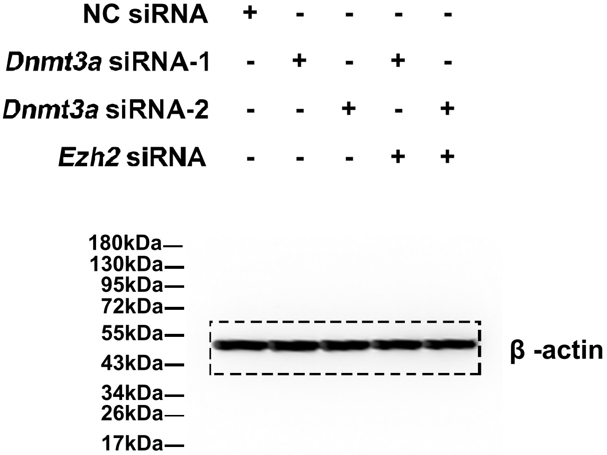

Original images (blots) of S3A Fig

Membranes scanned using Tanon 5500

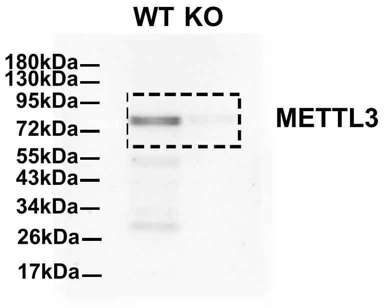

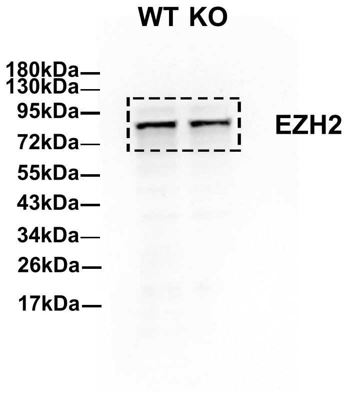

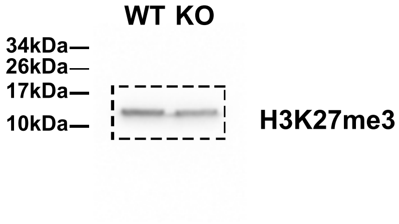

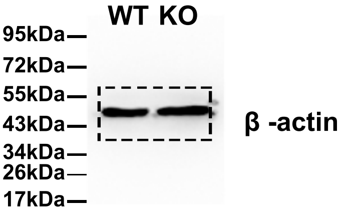

Original images (blots) of S3B Fig

Membranes scanned using Tanon 5500

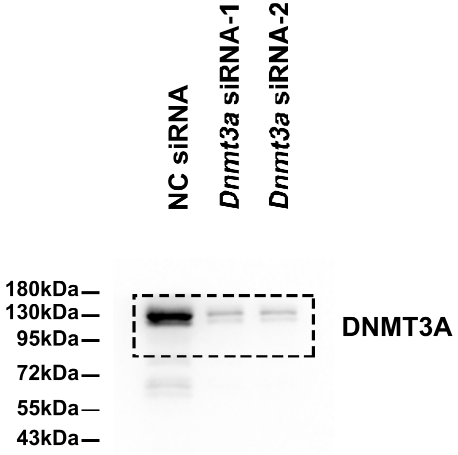

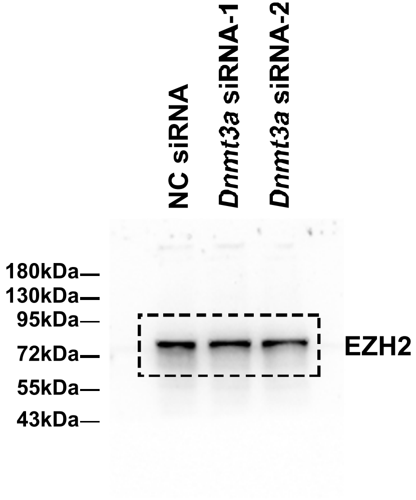

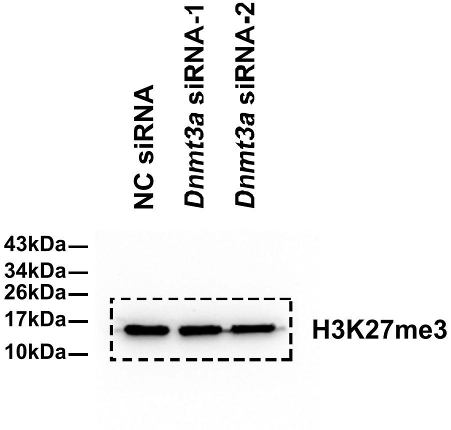

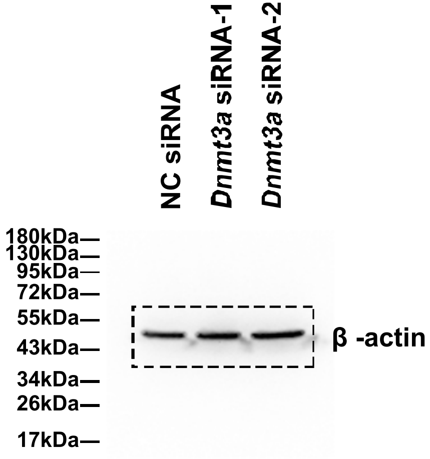

Original images (blots) of S4D Fig

Membranes scanned using Tanon 5500

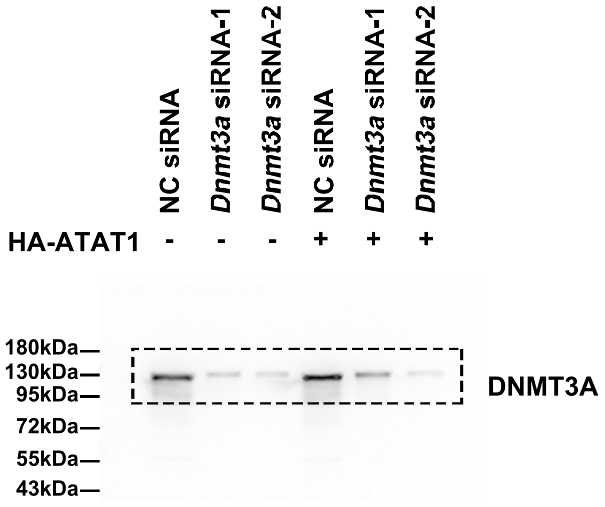

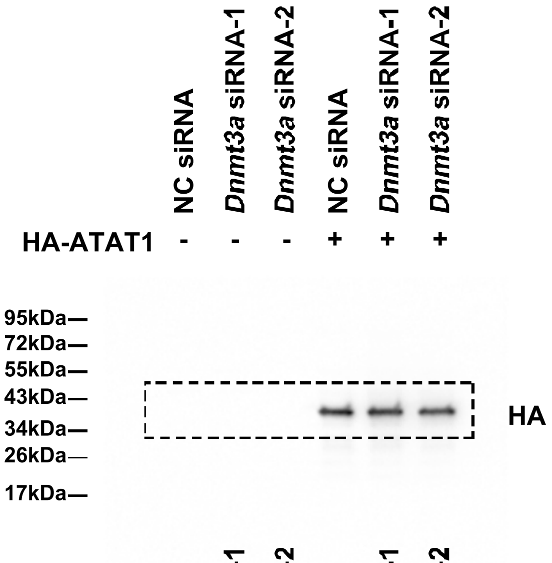

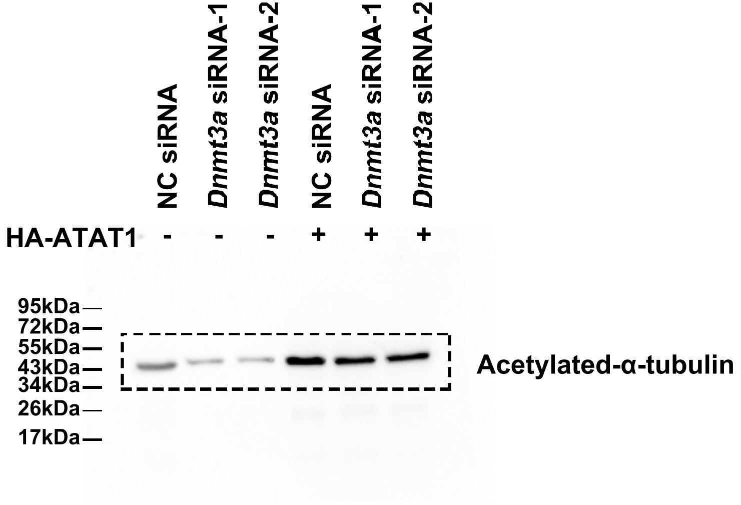

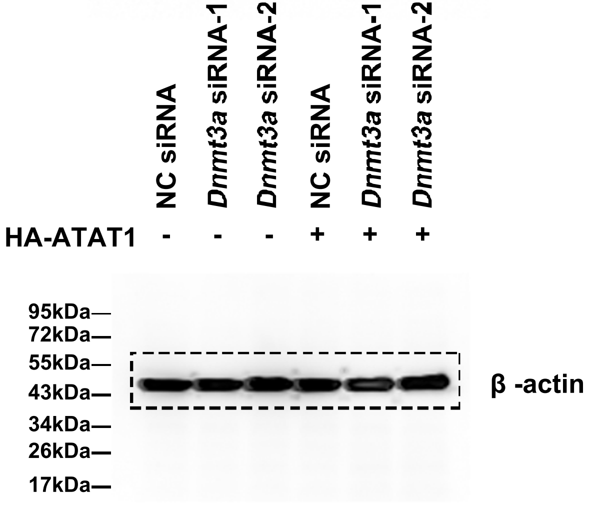

Supplement: S1 Raw images — (PDF) [file pbio.3002017.s009.pdf]
