# Supplementary material for: Loss of the m6A methyltransferase METTL3 in monocyte-derived macrophages ameliorates Alzheimer’s disease pathology in mice
Source: PLoS Biol. 2023 Mar 7;21(3):e3002017. doi: 10.1371/journal.pbio.3002017 (PMC9990945; doi:10.1371/journal.pbio.3002017)
Supplement: Attachment — Submitted filename: PLOS Biology response to reviewers.docx [file pbio.3002017.s011.docx]

**Revision Summary**

**1. Summary of major comments from Editor and Reviewers.**

Based on the comments from the reviewers, we have performed additional experiments and analysis. The details of our revision are summarized in the following **Table 1**.

**Table 1. Revision for major comments from the reviewers**

| **Key Questions** | | **Reviewers** | **Clarification on the original submission data** | **New experiments** | **New supporting data** |
| --- | --- | --- | --- | --- | --- |
| 1 | The authors should also analyze and exclude the function of METTL3 in microglia in their system. Whether the effects are mediated solely by monocyte-derived macrophages? | #1: Q2  #2: Q1 | N/A | We performed bone marrow (BM) transfer experiments and monocytes transplantation to examine the direct role of monocytes-derived macrophages in AD. In addition, we assessed the impact of enhanced migration of macrophages with *Mettl3* ablation in the progress of AD by CCR2 blocking in our system. | Our new data showed that METTL3-deficient bone marrow or monocytes transplantation could alleviate the symptoms of AD in mice (**Response Figures 1 and 8; Figs 1I, 1J, S1C and S4A**). The results showed that blocking of CCR2 signaling significantly impaired the transmigration of monocyte-derived macrophages into brain and aggravated the AD symptoms (**Response Figure 7; Figs 1K and S1D**). |
| 2 | Additional behavioral experiments need to be done to evaluate the role of METTL3 in monocyte-derived macrophages involved in AD progression. | #1: Q3  #2: Q2 | N/A | The Y maze test was performed to measure the spontaneous object and temporal order recognition memory of Aβ-induced WT or KO mice. | The results showed that *Mettl3* deficiency significantly improved the cognition function (**Response Figure 2A-C; Fig 1F-H**). |
| 3 | The authors should provide direct evidence that DNMT3A affects H3K27me3 catalyzed by EZH2 in Atat1 promoter region to promote ATAT1 expression. | #1: Q6 | N/A | We performed ChIP assays using EZH2 or H3K27me3 antibodies in DNMT3A knockdown cells to explore whether DNMT3A affect the occupy of EZH2 and H3K27me3 in *Atat1* promoter region. | The results exhibited that DNMT3A knockdown enhanced the binding capability of H3K27me3 and EZH2 in *Atat1* promoter region (**Response Figure 5C; Fig 5C**). Furthermore, we observed that double knockdown of both DNMT3A and EZH2 inhibited the reduced expression of ATAT1 induced by DNMT3A knockdown (**Response Figure 5D; Fig 5D**). |
| 4 | The authors should analyze the infiltration of macrophages in the CNS. | #1: Q7  #2: Q7 | N/A | Brain tissues from Aβ-induced AD mice were taken for analysis by flow cytometry. | We observed that the frequency of monocyte-derived macrophages (CD45+CD11b+F4/80+) from KO mice was increased significantly compared to that from WT mice (**Response Figure 6; Fig 6C**). |
| 5 | DNMT3A can affect the transcription of many genes. How do we know that all the downstream effects are mediated by Atat1? | #2: Q5 | N/A | We overexpressed ATAT1 in the DNMT3A knockdown cells. | The results showed that overexpression of ATAT1 inhibited the reduced alpha-tubulin acetylation (**Response Figure 11A; S4D Fig**), enhanced migration (**Response Figure 11B; Figs 6G and S4C**) and Aβ uptake (**Response Figure 11C; Fig 7D**) induced by DNMT3A knockdown. |
| 6 | Writing | #1: minor  #2: | N/A | The English Language in the manuscript has been edited by by a native English speaker. | N/A |

**2. Response to reviewers’ comments**

REVIEWS:

We would like to thank the reviewers for their supportive and constructive comments and are pleased that they find our work novel and interesting. We believe their questions and advice have helped us improve the manuscript.

Reviewer #1, Hua-Bing Li (note, this reviewer has signed the review):
In this manuscript, the authors investigated the role of m6A RNA methylation in monocyte-derived macrophage involved in the progression of AD. They reported that METTL3 ablation in myeloid cells improved cognition function in AD mouse model. Mechanistically, the authors demonstrated that METTL3 promoted the translation of DNMT3A mediated by YTHDF1. DNMT3A bounded at the promoter region of Atat1 and maintained its expression. Thus, loss of METTL3 resulted in decreased translation of DNMT3A and subsequently downregulated the expression of Atat1. The decreased expression of Atat1 led to reduced α-tubulin acetylation and enhanced monocyte-derived macrophage migration, Aβ clearance and relief of AD symptoms.

We thank the reviewer for a very comprehensive evaluation of our work.

There are several main concerns with this manuscript:

Major issues:

1. Introduction, page 3, line 65, the authors termed "blood-derived myeloid cells recruited by CNS damage" as " blood-derived microglia ". Recent studies show that microglia are tissue resident and originate in the yolk sac but not in the bone marrow from hematopoietic stem cells. Microglia and macrophages represent two related cell types involved in the brain pathology. The authors should replace the phrase " blood-derived microglia".

We thank the reviewer for this suggestion. We agree with the suggestion and have replaced the term “blood-derived microglia” with “blood-derived macrophages” scientifically in our revised manuscript (**page 3, lines 65-66**).

2. The author utilized using a Mettl3 conditional mouse line in combination with Lyz2-Cre driver to elucidate the role of m6A mRNA methylation in monocyte-derived macrophage involved in the progression of AD (Results, page 4, line 87). In this system, METTL3 is also deleted in microglia. Microglial phagocytosis ability is proved to be associated with Aβ deposition and affect AD progression. The authors should also analyze and exclude the function of METTL3 in microglia in their system.

Thank you for your comment. To exclude the function of METTL3 in microglia in our study. We need to examine the effects of METTL3 in microglia in our study by using a microglia-specific Cre (*Cx3cr1*-Cre) to delete METTL3 in microglia. The generation of microglia-specific METTL3 knockout mice would take us at least 1 year. Given the timeline for manuscript revision and the unavailability of the research material, we would greatly appreciate it if the reviewer allows us to not include these experiments in this manuscript. Instead, we performed bone marrow (BM) transplantation experiments to examine the direct role of monocytes-derived macrophage in AD. The wild type mice first received 9.0 Gy irradiation, then injected with bone marrow cells (1×10^7^) from WT or KO mice via the tail vein. Then the mice were subjected to Aβ treatment and tested by the Morris water maze task and Y maze test. We observed that the spatial learning and memory of KO BM-transferred mice were improved compared with WT BM-transferred mice (**Response Figure 1A and 1B; Figs 1I and S4A**). And the Aβ burden in the brains of mice receiving KO-BM transplantation was significantly reduced (**Response Figure 1C; S5A Fig**). These results suggested that monocyte-derived macrophages with METTL3 deficiency could certainly improve the cognitive function in mice.

**Response Figure 1** The wild type mice firstly received 9.0 Gy irradiation, then injected with bone marrow cells (1×10^7^) from WT or KO mice via the tail vein. Then the mice were subjected to Aβ treatment. Flow cytometry was used to analyse the monocyte-derived macrophages in brain (A). The learning and memory of mice were examined by Morris water maze task and Y-maze test (B). Representative fluorescence micrographs and quantitative analysis of Aβ in brains (C).

3. Figure1: The authors assessed the cognition function and locomotor behavior by Morris water maze test and open-field test respectively. They found that METTL3 ablation in myeloid cells improved the cognition function but not locomotor behavior. Additional behavioral experiments such as Y maze test need to be done to comprehensively evaluate the role of METTL3 in monocyte-derived macrophage involved in AD progression.

We thank the reviewer for the suggestion. We have now included the additional experiments. The Y maze test was performed to measure the spontaneous object and temporal order recognition memory of Aβ-induced WT or KO mice. The results showed that *Mettl3* deficiency significantly increased the frequency of correct alternations of mice in the Y maze test relative to control group (**Response Figure 2A-C; Fig 1F-H**), which indicated that METTL3 ablation improved the spatial working memory of mice.

**Response Figure 2** (A) Experimental design of the Y-maze test. (B) The spatial working memory of WT or KO mice induced by Aβ in the Y-maze test. Alternations were counted as percentage of ‘correct’ alternation/total entries. ‘Correct’ alternation means entry into all three arms on consecutive choices (i.e. ABC, BCA, or CAB, but not CAC, BAB, or ABA). (C) Representative Y-maze test plots for WT and KO mice induced by Aβ.

4. Fig. 2C, 2D and Fig. 3D: The expression of ATAT1 needs to be detected in both mRNA level and protein level.

We thank the reviewer for this comment. We have added Western blot analysis of ATAT1 protein in Fig. 2C, 2D and Fig. 3D, which is now shown in **Fig. 2C, 2D** and **Fig. 3E (Response Figure 3A-C)**.

**Response Figure 3** (A) Expression of METTL3 and ATAT1 in WT and KO BMDMs evaluated by immunoblotting. (B) Immunoblotting analysis of the expression of METTL3 and ATAT1 in the BMDMs transfected with NC siRNA or *Mettl3* siRNA. (C) Immunoblotting analysis of the indicated proteins in the BMDMs transfected with NC siRNA or *Dnmt3a* siRNA.

5. Fig.3: The authors picked nine of the downregulated m6A direct targets from their previous m6A-seq data set. The authors need to exhibit the ranking of these genes among all the differential targets in their m6A-seq data set, and to explain why they focus on these nine genes.

We thank the reviewer for this comment. We analysed our previous m6A-seq dataset GSE146140[36] in which *Mettl3* was knocked out specifically in myeloid cells. There are 70 m6A-downregulated genes in KO BMDMs (fold-change >4 and p value < 0.00001). We have now exhibited the ranking of the top 50 downregulated m6A target genes (**Response Figure 4; S2A Fig**). By performing Gene Ontology (GO) enrichment analysis for the m6A-downregulated genes, we found that the m6A-downregulated genes were mainly enriched in negative regulation of transcription containing the nine genes (**Fig 3A**). These results indicated that METTL3 may regulate the downstream genes through the nine genes. So we focused on the nine genes and further analysed their expression and correlation with downstream genes. We have added the detailed explanation in our revised manuscript (**page 6, lines 135-139**).

**Response Figure 4** The ranking of top 50 m6A-down regulated genes in KO BMDMs.

6. Fig 5: The author concluded that DNMT3A maintained the expression of ATAT1 by antagonizing H3K27me3 catalyzed by EZH2 in Atat1 promoter region. Whereas, the results in Fig5 cannot support their conclusion. They showed METTL3 deletion increased the abundance of H3K27me3 and EZH2 binding at the promoter region of Atat1. It is possible that METTL3 directly regulates the expression of EZH2 or the capability of EZH2 binding to the promoter region of Atat1. The authors should provide direct evidence that DNMT3A affects H3K27me3 catalyzed by EZH2 in Atat1 promoter region to promote ATAT1 expression.

We thank the reviewer for this comment. To explore the mechanism underlying the regulation of ATAT1 by DNMT3A, we first analysed the expression of EZH2 and H3K27me3 in WT and KO BMDMs. The results showed that METTL3 did not affect the abundance of EZH2 and H3K27me3 (**Response Figure 5A; S3A Fig**). We also detected the expression of EZH2 and H3K27me3 in DNMT3A knockdown cells and demonstrated that the DNMT3A did not affect the EZH2 and H3K27me3 levels (**Response Figure 5B; S3B Fig**). These results indicated that METTL3 and DNMT3A did not regulate the expression of EZH2. Next, we performed ChIP assays using EZH2 or H3K27me3 antibodies in DNMT3A knockdown cells to explore whether DNMT3A affects the occupation of EZH2 or H3K27me3 in the *Atat1* promoter region. The results showed that DNMT3A knockdown enhanced the binding capability of H3K27me3 and EZH2 in the *Atat1* promoter region (**Response Figure 5C; Fig 5C**). Previous results have demonstrated that knockdown of DNMT3A inhibited the expression of ATAT1 (**Fig 3D and 3E**). Furthermore, we observed that double knockdown of both DNMT3A and EZH2 inhibited the reduced expression of ATAT1 induced by DNMT3A knockdown (**Response Figure 5D; Fig 5D**). These data suggested that DNMT3A maintained the expression of ATAT1 by antagonizing H3K27me3 catalyzed by EZH2 in the *Atat1* promoter region.

**Response Figure 5** (A) Immunoblotting analysis of the indicated proteins in the WT and KO BMDMs. (B) Immunoblotting analysis of the indicated proteins in the BMDMs transfected with NC siRNA or *Dnmt3a* siRNA. (C) ChIP analysis of H3K27me3 or EZH2 at the *Atat1* promoter in the DNMT3A knockdown BMDMs. (D) The expression of DNMT3A, EZH2 and ATAT1 was measured by qRT-PCR and Western blots in the BMDMs transfected with NC, *Dnmt3a* or *Dnmt3a* and *Ezh2* siRNAs.

7. Fig 6A: The authors should analyze both percentage and absolute numbers of macrophages in the CNS through flow cytometry.

Thank you for this suggestion. We have now performed the required experiments. Brain tissues from Aβ-induced AD mice were taken for analysis by flow cytometry. We observed that the frequency of macrophages (CD45+CD11b+F4/80+) from the KO mice was increased significantly compared to that from the WT mice (**Response Figure 6; Fig 6C**). Both the percentage and absolute numbers of macrophages in brains were analysed statistically (**Response Figure 6; Fig 6C**).

**Response Figure 6** Flow cytometry analysis of brains from Aβ-induced WT or KO mice. And the percentages and absolute numbers of macrophages in brains were quantified.

8. Fig 6: The authors demonstrated that METTL3 depletion enhanced transmigration of brain-infiltrating macrophage. The authors should assess the impact of enhanced migration of macrophages on the progress of AD by using CCR2 blocking antibody in their system.

We thank the reviewer for this suggestion. Owing to the unavailability of CCR2 blocking antibody, we alternatively investigated the role of enhanced migration of macrophages in the progress of AD with the pharmacological blockade of CCR2 signaling, PF-4136309, a CCR2 antagonist that has been shown to specifically interact with CCR2 (Ratnayake et al., 2021; Xue et al., 2011). The results showed that blocking of CCR2 signaling significantly impaired the transmigration of monocyte-derived macrophages into the brain and aggravated the AD symptoms (**Response Figure 7; Figs 1K and S1D**). These results suggested that the enhanced transmigration of monocyte-derived macrophages induced by METTL3 deficiency could improve the symptoms of AD.

**Response Figure 7** (A) Flow cytometry analysis of brain from Aβ-induced WT or KO mice injected with CCR2 antagonist PF-4136309 (2 mg/kg). (B) The learning and memory of Aβ-induced WT or KO mice injected with CCR2 antagonist PF-4136309 (2 mg/kg) were assessed by MWM task and Y-maze test.

Minor issues:

1. Statistical methods: The pairwise comparisons should be analyzed for significance with nonparametric (Mann-Whitney) tests rather than the t-test, because of the small numbers of repeats and the non-normally distributed data points where they are shown.

We thank the reviewer for this comment. Following the reviewer’s suggestion, we have modified the statistical methods in our manuscript accordingly.

2. I would suggest the authors to have the manuscript language edited by a native English speaker/professional.

We thank the reviewer for this comment. The language in our manuscript has been edited by a native English speaker.

Reviewer #2:

The paper satisfies all the requirements for publication and can be accepted as it stands

We thank the reviewer for the positive comments.

Reviewer #3:

Yin et al. documented a long chain of observations after cell-type-specific Mettl3 deletion with the Lyz2-Cre driver. Specifically,

1. Mettl3 deletion with the Lyz2-Cre driver -->
2. Reduced m6A modification of Dnmt3a mRNA in monocyte-derived macrophages -->
3. Reduced YTHDF1 binding to Dnmt3a mRNA -->
4. Reduced translation of Dnmt3a mRNA to DNMT3A protein -->
5. Reduced antagonization of EZH2 by DNMT3A, increased trimethylation of histone H3 in Atat1 promoter region -->
6. Reduced transcription of Atat1 -->
7. Reduced acetylation of alpha-tubulin -->
8. Enhanced infiltration of monocyte-derived macrophages into the brain -->
9. Enhanced A-beta clearance -->
10. Improved cognition in A-beta based AD model

The strength of the study is its potential significance in treating AD. Another strength is in proposing very specific hypotheses and in conducting studies on many of the downstream pathways. Unfortunately, for the same reason, it's an enormous burden to prove that each step in this very long chain of events is direct, specific, and causal. I will list these concerns below.

We thank the reviewer for the positive and helpful comments.

1) There are studies showing that Lyz2 is also expressed in microglia in addition to myeloid cells. It's also expressed in other non-macrophage blood cells. How are the authors sure that the effects are mediated solely by monocyte-derived macrophages?

Thanks for pointing this out. We appreciate that we cannot formally exclude the impact of other myeloid cells from *Lyz2*^Cre^*Mettl3*^fl/fl^ mice. Alternatively, we can examine the direct role of monocyte-derived macrophages by transferring CD115^+^ monocytes isolated from the bone marrow of WT or KO C57BL/C mice into Aβ-induced wild-type mice. In the manner, we can examine whether the monocytes-derived macrophage is directly associated with Aβ deposition and AD progression. The results showed that the spatial learning and memory of mice with KO monocytes transplantation were improved compared with those receiving WT monocytes, as demonstrated by the Morris water maze task and Y maze test (**Response Figure 8A and 8B; Figs 1J and S1C**). And the Aβ burden in the brain of mice receiving KO monocytes was also significantly reduced (**Response Figure 8C; S5B Fig**). These results indicated that monocyte-derived macrophages with METTL3 ablation could alleviate symptoms of AD.

**Response Figure 8** (A) The purity of isolated monocytes was checked by flow cytometry. (B) The spatial learning and memory of mice with WT or KO monocytes transplantation were examined by Morris water maze task and Y-maze test. (C) Representative fluorescence micrographs and quantitative analysis of the brains from Aβ-induced mice transferred with WT and KO monocytes.

2) For AD-related cognitive impairments, only water maze was used. In the water maze test, only probe trial data was used. There's no data on learning. This is very limited, especially because the whole story relies on the claim that the treatment reduces cognitive impairments.

We thank the reviewer for this comment. We have added the learning data (**Response Figure 9; Fig 1A**). In addition, the Y maze test required by reviewer #1 was performed to measure the spontaneous object and temporal order recognition memory of Aβ-induced WT or KO mice. The results showed that *Mettl3* deficiency significantly increased the frequency of correct alternations of mice in the Y maze test relative to control group (**Response Figure 2A-C; Fig 1F-H**). These results indicated that METTL3 ablation could improve the cognition function in AD mice.

**Response Figure 9** Escape latencies of WT or KO mice in Morris water maze task on training days were recorded.

3) Fig 3B shows very small and non-significant changes in Dnmt3a (p=0.80)

Thanks for pointing this out, the mRNA expression of the *Dnmt3a* was indeed not altered significantly in KO BMDMs compared with that in WT BMDMs (**Figs 3B and 4B**). Followingly, we found that the DNMT3A protein level was significantly reduced in KO BMDMs (**Fig 4B and 4C**), which suggested that METTL3 regulated DNMT3A protein levels rather than mRNA expression.

4) In Fig 4G, reduced DNMT3A level is not clear. There's no statistics either. If the argument is reduced translation, is the mRNA level normal to begin with in this experiment?

We thank the reviewer for this comment. We have now added a statistical image to Figure 4G. The results showed that the expression of *Dnmt3a* WT, *Dnmt3a* Mut1, *Dnmt3a* Mut2 or *Dnmt3a* Mut1/2 was significantly different (**Response Figure 10A; Fig 4G**). We also examined the mRNA levels by qRT-PCR and demonstrated that the mRNA levels of *Dnmt3a* WT, *Dnmt3a* Mut1, *Dnmt3a* Mut2 or *Dnmt3a* Mut1/2 were almost equal (**Response Figure 10B; Fig 4H**). These results supported that m6A modification mainly regulated the DNMT3A translation rather than the expression of *Dnmt3a* mRNA.

**Response Figure 10** (A) The expression of DNMT3A WT, DNMT3A mut1, DNMT3A mut2 or DNMT3A mut1/2 was measured by Western blot. The normalized intensity of DNMT3A WT, DNMT3A mut1, DNMT3A mut2 or DNMT3A mut1/2 was quantified. (B) qRT-PCR analysis of the expression of *Dnmt3a* WT, *Dnmt3a* Mut1, *Dnmt3a* Mut2 or *Dnmt3a* Mut1/2.

5) DNMT3A can affect the transcription of many genes. How do we know that all the downstream effects are mediated by Atat1?

We thank the reviewer for this comment. To confirm whether the downstream effects caused by DNMT3A in our study are mediated by ATAT1, we overexpressed ATAT1 in DNMT3A knockdown cells. The results showed that overexpression of ATAT1 inhibited the reduced alpha-tubulin acetylation (**Response Figure 11A; S4D Fig**), enhanced migration (**Response Figure 11B; Figs 6G and S4C**) and Aβ uptake (**Response Figure 11C; Fig 7D**) induced by DNMT3A knockdown.

**Response Figure 11** (A) Immunoblotting analysis of the indicated proteins in HA-ATAT1-overexpressing cells transfected with NC siRNA or *Dnmt3a* siRNA. (B) Transwell assays were performed to determine the cell migration of HA-ATAT1-overexpressing cells transfected with NC siRNA or *Dnmt3a* siRNA. Scale bars: 30 µm. (C) Representative fluorescence micrographs and quantitative analysis of Aβ uptake in HA-ATAT1-overexpressing cells transfected with NC siRNA or *Dnmt3a* siRNA. Scale bars: 10 µm.

6) Acetylation of alpha-tubulin has many functional consequences. How do we know that infiltration of monocyte-derived macrophages into the brain is responsible for the A-beta and behavioral phenotype?

We thank the reviewer for raising this concern. To test whether monocyte-derived macrophages into the brain is responsible for the A-beta and behavioral phenotype, we transferred CD115+ monocytes isolated from the bone marrow of WT or KO mice into Aβ-induced wild-type mice. Then we examined the A-beta burden in brain and behavior of Aβ-induced AD mice. The results showed that the spatial learning and memory of mice with KO monocytes transferred were improved compared with those receiving WT monocytes (**Response Figure 8A and 8B; Figs 1J and S1C**). And the Aβ burden in brains of mice with KO monocytes transferred was also significantly reduced (**Response Figure 8C; S5B Fig**). These results indicated that monocyte-derived macrophages with METTL3 ablation could alleviate symptoms of AD.

7) There is no direct evidence of enhanced infiltration of monocyte-derived macrophages into the brain.

Thanks for pointing this out. A recent study has demonstrated that peripheral monocytes contributed to a high fraction of macrophages in the brain of aged AD mice by using a genetic fate-mapping approach (Yan et al., 2022). In our study, the brain tissues from Aβ-induced AD mice were taken for analysis by flow cytometry. We observed that the frequency of CD45+CD11b+F4/80+ cells from KO mice was increased significantly compared to that from WT mice (**Response Figure 6; Fig 6C**). In addition, we transferring CD115+ monocytes isolated from the bone marrow of WT or KO C57BL/C mice (CD45.2) into Aβ-induced wild-type mice (CD45.1). Brain tissue from mice were taken for analysis by flow cytometry. We observed that the frequency of monocyte-derived macrophages (CD45.2+CD11b+F4/80+) in brain from KO monocytes-transferred mice was increased significantly compared to that receiving WT monocytes (**Response Figure 12; S4B Fig**). These results indicated that METTL3 deficiency enhances the infiltration of monocyte-derived macrophages into the brain.

**Response Figure 12** Flow cytometry analysis of brain from Aβ-induced mice (CD45.1) transferred with WT and KO monocytes (CD45.2). And the percentage and absolute numbers of macrophages in brain were quantified.

8) In Fig 6B, the Y axis is not explained. What does this "%" mean?

We thank the reviewer for this comment. We have included text in figure legends to explained the "%" mean in **Fig 6B**. It means the percentage of CD45^+^IBA1^+^ cell in the brains of 24-month-old mice.

9) What is the normal "good" function of m6A in monocyte-derived macrophages? How do they become maladaptive in AD?

Thanks for pointing this out. Although the role of mRNA m6A methylation has been increasingly understood in recent years, some significant challenges remain unresolved. The study on m6A in monocyte-derived macrophages is just beginning. Numerous questions about macrophage polarization and their roles in health and disease remain incompletely answered. A previous report demonstrating that METTL3 significantly attenuates the LPS-induced inflammatory response in macrophages (Wang et al., 2019), but seemingly contradict another study showing that METTL3 facilitates M1 macrophage polarization (Liu et al., 2019). Recently, our study and other group showed that depletion of METTL3 or METTL14 in macrophage enhanced cancer progression (Dong et al., 2021; Tong et al., 2021; Yin et al., 2021). So we think the roles of m6A in monocyte-derived macrophages might be different depending on the local environment.

Collectively evidences showed that changes in the macrophage differentiation, polarization, re-polarization, and activation in the local milieu can play a decisive role in the pathogenesis of a wide variety of autoimmune and inflammatory diseases (Shapouri-Moghaddam et al., 2018). The monocyte-derived macrophages is more likely an external participant in AD. Monocyte-derived macrophages sense the local environment signal and participate in progression of AD. However, we don’t know how the METTL3 was regulated in the progression of AD. A previous study also showed that the expression of the m6A methyltransferase METTL3 was elevated in AD mice (Han et al., 2020). We will further investigate the mechanism underlying METTL3 regulation in AD. The upstream regulation of METTL3 remained to be explored. We have added the discussion in our manuscript (**page 12, lines 285-286**).

10) M6A is known to be essential in cell differentiation, which has been shown in many cell types. Does Mettl3 deletion affect macrophage differentiation?

We thank the reviewer for this comment. We performed experiments and demonstrated that METTL3 deficiency could not significantly alter the expression of monocytes-derived macrophage cell markers (CD11b and F4/80) (**Response Figure 13; S1A Fig**). However, the function of macrophage was influenced by METTL3 depletion. Our previous study has shown that *Mettl3* deletion enhanced M2 macrophage polarization and accelerated tumor growth and metastasis (Yin et al., 2021).

**Response Figure 13** Flow cytometry of the surface CD11b and F4/80 expression of primary cultured bone marrow derived macrophages.

Overall, the manuscript is not clearly written. In one short paragraph, "blood-derived microglia", "blood-derived monocytes", and "monocyte-derived macrophages" were all used. And in most other places, "bone marrow derived macrophages" were used. Do these four mean the same thing? Also, is "blood-derived microglia" even correct?

Thanks for pointing this out. We apologize for the confusion. Firstly, microglia originating from the yolk sac are the resident macrophages of the central nervous system. Microglia is different from "blood-derived microglia", "blood-derived monocytes", and "monocyte-derived macrophages", which originate from hematopoietic stem cells. In previous studies, "blood-derived microglia" and "monocyte-derived macrophages" were both used to represent recruited macrophages in brain by many researchers owing to controversial origin of microglia. We think the two phrases represent almostly the same thing. In addition, "blood-derived monocytes" could differentiate into "monocyte-derived macrophages". We have modified the text scientifically for well readability (**page 3, lines 62-66**).

The bone marrow derived macrophages (BMDMs) present an ideal *in vitro* model to understand the mechanisms controlling the functions of macrophages. In our study, we also used BMDMs as the research object to study molecular mechanism controlled by METTL3.

Overall, there is no good logical flow in the writing. Many arguments seem to be non sequitur, e.g., the first and second paragraphs of "Discussion". In the "Introduction", the fact that this has never been studied before is not a good enough argument for studying it. There must be better reasons, but they were not explained. Too many things have never been studied before because they may not be worth studying.

Thanks for pointing this out. We agree with this suggestion and have modified the text throughout the manuscript (**page 2, lines 45-47; page 3, lines 68-69; page 10, lines 238-241; pages 10-11, lines 249-250**).

Despite these serious concerns, I still think it's worth publishing these observations along with careful discussions of alternative interpretations.

.

__________________________________________________
In compliance with data protection regulations, you may request that we remove your personal registration details at any time. (Use the following URL: https://www.editorialmanager.com/pbiology/login.asp?a=r). Please contact the publication office if you have any questions.

Dong, L., Chen, C., Zhang, Y., Guo, P., Wang, Z., Li, J., Liu, Y., Liu, J., Chang, R., Li, Y.*, et al.* (2021). The loss of RNA N(6)-adenosine methyltransferase Mettl14 in tumor-associated macrophages promotes CD8(+) T cell dysfunction and tumor growth. Cancer Cell *39*, 945-957.e910.

Han, M., Liu, Z., Xu, Y., Liu, X., Wang, D., Li, F., Wang, Y., and Bi, J. (2020). Abnormality of m6A mRNA Methylation Is Involved in Alzheimer's Disease. Front Neurosci *14*, 98.

Liu, Y., Liu, Z., Tang, H., Shen, Y., Gong, Z., Xie, N., Zhang, X., Wang, W., Kong, W., Zhou, Y., and Fu, Y. (2019). The N(6)-methyladenosine (m(6)A)-forming enzyme METTL3 facilitates M1 macrophage polarization through the methylation of STAT1 mRNA. Am J Physiol Cell Physiol *317*, C762-c775.

Ratnayake, D., Nguyen, P. D., Rossello, F. J., Wimmer, V. C., Tan, J. L., Galvis, L. A., Julier, Z., Wood, A. J., Boudier, T., Isiaku, A. I.*, et al.* (2021). Macrophages provide a transient muscle stem cell niche via NAMPT secretion. Nature *591*, 281-287.

Shapouri-Moghaddam, A., Mohammadian, S., Vazini, H., Taghadosi, M., Esmaeili, S. A., Mardani, F., Seifi, B., Mohammadi, A., Afshari, J. T., and Sahebkar, A. (2018). Macrophage plasticity, polarization, and function in health and disease. J Cell Physiol *233*, 6425-6440.

Tong, J., Wang, X., Liu, Y., Ren, X., Wang, A., Chen, Z., Yao, J., Mao, K., Liu, T., Meng, F. L.*, et al.* (2021). Pooled CRISPR screening identifies m(6)A as a positive regulator of macrophage activation. Science advances *7*.

Wang, J., Yan, S., Lu, H., Wang, S., and Xu, D. (2019). METTL3 Attenuates LPS-Induced Inflammatory Response in Macrophages via NF-κB Signaling Pathway. Mediators Inflamm *2019*, 3120391.

Xue, C. B., Wang, A., Han, Q., Zhang, Y., Cao, G., Feng, H., Huang, T., Zheng, C., Xia, M., Zhang, K.*, et al.* (2011). Discovery of INCB8761/PF-4136309, a Potent, Selective, and Orally Bioavailable CCR2 Antagonist. ACS Med Chem Lett *2*, 913-918.

Yan, P., Kim, K. W., Xiao, Q., Ma, X., Czerniewski, L. R., Liu, H., Rawnsley, D. R., Yan, Y., Randolph, G. J., Epelman, S.*, et al.* (2022). Peripheral monocyte-derived cells counter amyloid plaque pathogenesis in a mouse model of Alzheimer's disease. J Clin Invest *132*.

Yin, H., Zhang, X., Yang, P., Zhang, X., Peng, Y., Li, D., Yu, Y., Wu, Y., Wang, Y., Zhang, J.*, et al.* (2021). RNA m6A methylation orchestrates cancer growth and metastasis via macrophage reprogramming. Nat Commun *12*, 1394.
